# On Infanticide

**Published:** 1803-05-01

**Authors:** 


### *To the Editors of the Medical and Physical Journal.*

Gentlemen,

As cases of supposed Infanticide frequently occur, I beg leave to call the attention of your readers to Dr. William Hunter's excellent paper on that subject, in the 6th volume of the "Medical Observations and Inquiries." As the work, from its contents, is not likely to be in the hands of the generality of readers, and is now rather scarce, it is sincerely to be wished that this paper on the *Uncertainty of the signs of murder in the case of bastard children*, were republished by itself, as it clearly and satifactorily proves that the commonly supposed signs of murder in those cases, such as swimming of the lungs, blackness in the face, &c. &c. are so very fallacious and uncertain, that not the the least dependence can be placed upon them, and that they are frequently found to arise from other causes. With respect to the concealment of a still-born child, or one which dies soon after birth (which frequently happens) in an unmarried woman, it cannot but be allowed to be highly natural, as it arises from an unconquerable sense of shame, from a desire to preserve her character, and from want of resolution to meet and avow infamy.

